# Fecal DNA Metabarcoding Reveals the Diet of Asian Elephant in China During the Dry Season: Implications for Adaptation to Habitat Resources and Conservation

**DOI:** 10.1002/ece3.72398

**Published:** 2025-11-08

**Authors:** Qiang Guo, Wenping Zhang, Xu Li, Bin Wang, Chaoyong Xiong, Yuan Tian, Tingting Luo, Weibin Wang, Jielong Zhou

**Affiliations:** ^1^ College of Biological Science and Food Engineering/Key Laboratory of Conserving Wildlife With Small Populations in Yunnan/Key Laboratory of Forest Resources Conservation and Utilization in the Southwest Mountains of China, Ministry of Education Southwest Forestry University Kunming China; ^2^ Key Laboratory of Monitoring Biological Diversity in Minshan Mountain of National Park of Giant Pandas at Mianyang Normal University of Sichuan Province, College of Life Science Mianyang Normal University Mianyang China; ^3^ Asian Elephant Rescuing and Breeding Center Management and Protection Bureau of Yunnan Xishuangbanna National Nature Reserve Xishuangbanna Dai Autonomous Prefecture China; ^4^ Management and Protection Bureau of Yunnan Nangunhe National Nature Reserve Lincang China

**Keywords:** Asian elephant, dietary composition, dietary diversity, DNA metabarcoding, habitat restoration

## Abstract

The Asian elephant ( *Elephas maximus* ) is a flagship species of the tropical forest ecosystem in Asia, playing a crucial role in maintaining ecological stability. Investigating the dietary composition of Asian elephants is essential for developing effective conservation and management strategies. In this study, a total of 107 fecal samples from different Asian elephant populations in China were analyzed using chloroplast *rbcL* DNA metabarcoding to systematically examine the dietary composition and diversity of the species. The results show that the foraged resources of the Asian elephant encompass eight classes, 43 orders, 77 families, and 154 genera. At the order level, Poales, Fabales, Rosales, and Zingiberales have the highest proportions, whereas at the family level, Poaceae, Fabaceae, Cyperaceae, Moraceae, and Musaceae dominate. Diversity and ecological niche width analyses indicate that there are differences among populations, with geographical variations in diet that are likely related to the availability of habitat resources. This study reveals the dietary composition and differences among different populations of Asian elephants, providing important scientific evidence and practical guidance for optimizing the food structure of captive populations and the development of food resource bases.

## Abstract 2

This study utilized DNA metabarcoding technology to analyze the dietary information of different Asian elephant populations in China, revealing differences in dietary composition and diversity. The results showed that the diet of Asian elephants primarily consists of Poaceae, Fabaceae, Cyperaceae, Musaceae, and Moraceae plants, with significant geographical differences. This indicates that Asian elephants exhibit flexible feeding strategies, and their dietary structure is influenced by habitat plant resources and environmental conditions. Additionally, the diet of captive populations, which is dominated by a single plant species, may have adverse effects on their health.

## 1 Introduction

**FIGURE 1:**
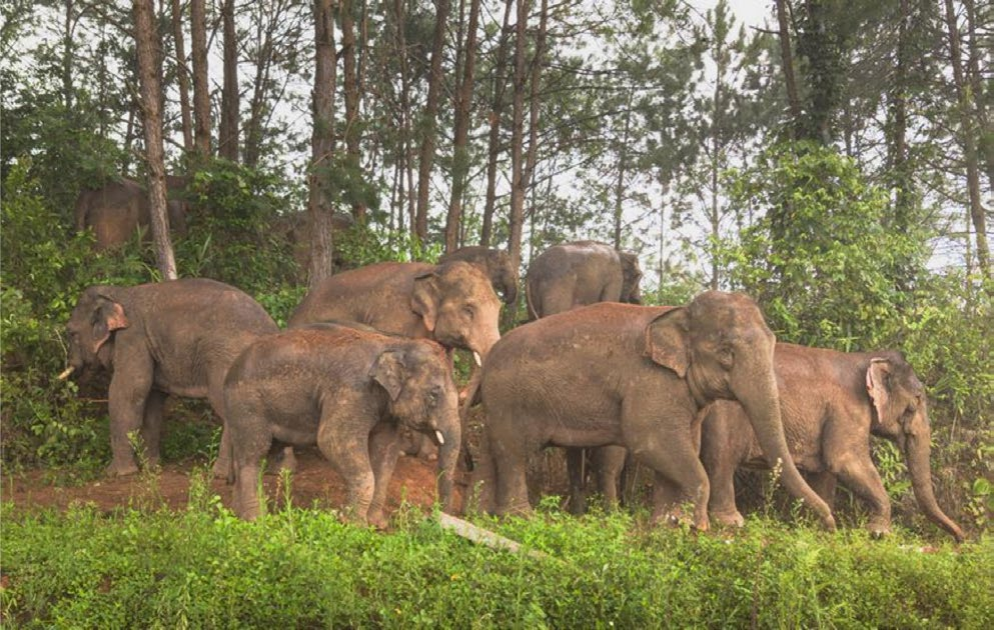
Photographs of wild Asian elephants in China. The photo was taken by Chenhao Zhou from the Asian Elephant Conservation and Management Center of Xishuangbanna Dai Autonomous Prefecture in Jinghong city, Xishuangbanna Dai Autonomous Prefecture, Yunnan Province, china.

**FIGURE 2:**
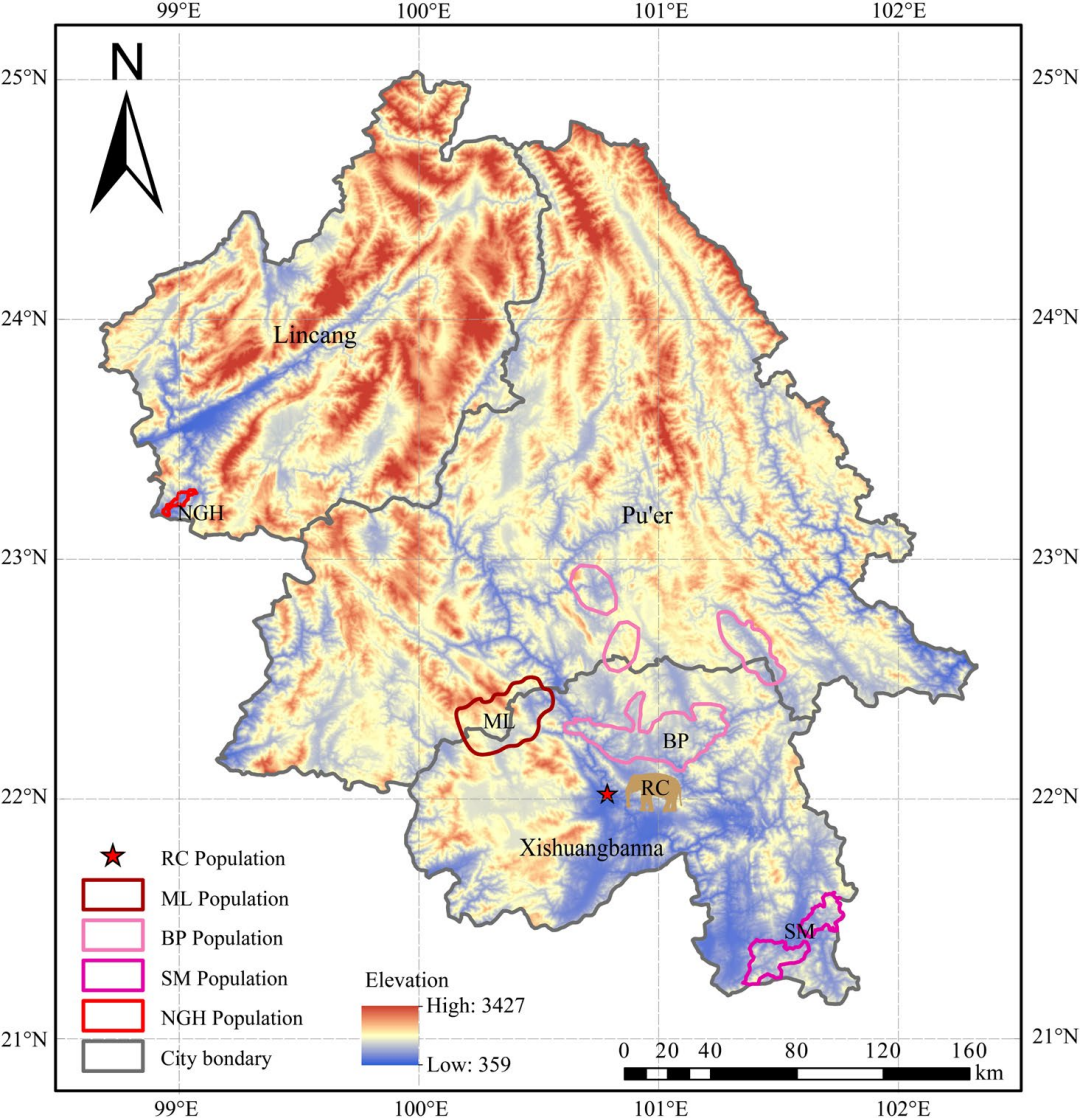
The locations of the Asian elephant fecal sample collection in Yunnan Province, China. The five‐pointed star marker represents the Xishuangbanna Asian Elephant Rescue and Breeding Center population (RC, *n* = 11). The areas marked with different colors indicate the distribution ranges of various wild populations. BP: Banna‐Pu'er population (*n* = 60), SM: Shangyong‐Mengla population (*n* = 8), ML: Menghai‐Lancang population (*n* = 9), NGH: Nangunhe population (*n* = 19).

The Asian elephant ( *Elephas maximus* , Figure 1), as the flagship and umbrella species of tropical forest ecosystems in Asia, is a national first‐level important protected wild animal in China and is listed as an Endangered (EN) species by the International Union for Conservation of Nature (IUCN) Red List (Chen et al. 2021; Luo et al. 2022; Su et al. 2020). Protecting Asian elephants not only protects other sympatric species but also contributes to the conservation of biodiversity in the tropical moist and tropical dry broadleaf forests (Sun et al. 2021). At present, only approximately 300 wild Asian elephants in China are extant and are distributed in Xishuangbanna Dai Autonomous Prefecture, Pu'er City, and Lincang City in Yunnan Province (Figure 2), and they can be classified into four genetic populations, namely the Banna‐Pu'er, Shangyong‐Mengla, Menghai‐Lancang, and Nangunhe populations (Sun et al. 2021; Yu et al. 2023).

To better protect Asian elephants, the Chinese government has continuously strengthened the construction of both in situ and ex situ conservation efforts, such as setting up the Xishuangbanna Asian Elephant Rescue and Breeding Center. Through the joint efforts of relevant Chinese authorities and local communities, the population of Asian elephants has gradually increased (Cao et al. 2023; Chen et al. 2023). Meanwhile, the expansion of forest canopy coverage has reduced the availability of food resources in the forest understory (Yu et al. 2023), prompting Asian elephants to move outside reserve areas to forage, which has led to agricultural losses and intensified human–elephant conflict (Campos‐Arceiz et al. 2021; Peng et al. 2023; Wang et al. 2021). The human–elephant conflict has become a widespread challenge across the range countries of Asian elephants (Basari et al. 2025; Shahid et al. 2025; Supanta et al. 2025), highlighting the urgent need to promote harmonious coexistence through scientific approaches and effective management strategies. To address these issues, the Chinese government has begun establishing ecological corridors and food resource bases (Li et al. 2023; Zhang et al. 2024). Nevertheless, inappropriate food supplementation may impact digestion rates and increase the risk of disease transmission (Couch, Wise, et al. 2021; Putman and Staines 2004). Therefore, studying the dietary structure of Asian elephants is of great significance for optimizing food resource allocation for captive populations and supporting the construction of ecological corridors and food resource bases.

At present, the lack of a comprehensive and integrated dataset on the dietary composition and preferences of Asian elephants in China has become an obstacle to formulating effective population conservation strategies. Previous studies on the diets of Asian elephants in China have primarily used direct observation, food trace analysis, fecal microanalysis, and DNA metabarcoding of a limited number of individuals (Chen et al. 2006; Jiang et al. 2019; Peng et al. 2023). Although these studies have provided valuable insights into the dietary habits of Asian elephants, they focused only on certain populations and thus lack comprehensive coverage. Additionally, understanding the diet of endangered wildlife also provides invaluable insights for assessing habitat quality, population dynamics, and ecosystem functions in addition to enhancing our understanding of their forage composition and survival strategies (Fu et al. 2024; Hacker et al. 2021). However, the differences in dietary composition among Asian elephant populations in China and their relationship with habitat heterogeneity remain insufficiently understood.

This study aims to explore the dietary composition of Asian elephants by collecting fecal samples from different populations and applying DNA metabarcoding. The results are expected to contribute to a better understanding of the foraging ecology of Asian elephants and provide a valuable reference for the effective development of future conservation and management strategies.

## 2 Materials and Methods

### 2.1 Dung Sample Collection

To ensure sample diversity, we collected fresh fecal samples from different wild populations and the Xishuangbanna Asian Elephant Rescue and Breeding Center (RC) in Yunnan, China (Figure 2). From January to April 2023, we collected fresh dung samples from different populations. For each sample, the surface portion was used for microsatellite and mitochondrial DNA analyses, whereas the inner portion was used for DNA metabarcoding sequencing to investigate dietary composition. All samples were immediately frozen after collection and transported to the laboratory for further processing. In total, 107 individual samples were identified for use in this study, including 60 Banna‐Pu'er (BP) samples, eight Shangyong‐Mengla (SM) samples, nine Menghai‐Lancang (ML) samples, 19 Nangunhe (NGH) samples, and 11 RC samples (Figure 2 and Table S1). The RC population is kept in a confined area at the rescue center, where individuals are regularly fed by humans rather than foraging freely in the wild.

### 2.2 Diet DNA Extraction and DNA Metabarcoding Sequencing

The total DNA was extracted from fecal samples of Asian elephant using the DNeasy Plant Pro Kit (Qiagen, Germany), according to the manufacturer's instructions. The concentration and purity of DNA were measured using a NanoDrop 2000 spectrophotometer (Thermo Fisher Scientific, America). Using the extracted DNA as a template, the pair of primers rbcL2_F (5′‐YGATGGACTTACNAGTCTTGATCGTTACA‐3′) and rbcL2_R (5′‐GNCCATAYTTRTTCAATTTATCTCTTTCAACTTGGATNCC‐3′) were used to amplify the chloroplast *rbcL* gene (ribulose‐bisphosphate carboxylase gene) region (Coghlan et al. 2020). Additionally, a negative control without the DNA template was set up to detect potential contamination, which showed no detectable contamination. For the polymerase chain reaction (PCR), we used 15 μL Phusion High‐Fidelity PCR Master Mix, 0.2 μM primers, and 10 ng of DNA template, and adjusted with ddH_2_O to a total reaction volume of 25 μL. The PCR amplification procedure was the initial denaturation temperature of 98°C for 1 min, followed by 30 cycles of denaturation at 98°C for 10 s, annealing at 50°C for 30 s and extension at 72°C for 30 s, and a final extension at 72°C for 5 min. The PCR products were detected by electrophoresis on a 2% agarose gel, purified using Agencourt AMPure XP beads (Beckman Coulter, USA), and quantified using the Qubit 3.0 DNA detection kit (Thermo Fisher Scientific, USA). The purified and quantified PCR products were pooled in equal volumes and then used for DNA library construction with the TruSeq Nano DNA LT Library Prep Kit for Illumina. The concentration of the DNA was measured using a NanoDrop 2000 spectrophotometer (Thermo Fisher Scientific, America), followed by pooling at equimolar concentrations. Next‐generation sequencing was performed by Novogene (Beijing, China) on the Illumina NovaSeq 6000 platform (2 × 150 bp paired‐end reads).

### 2.3 Dietary Analysis Using Plant Chloroplast *rbcL* Metabarcoding

After sequencing, the sample data were demultiplexed on the basis of the barcode, and barcode sequences were removed. The reads from each sample were merged using FLASH (Version 1.2.11), generating raw reads (Magoc and Salzberg 2011). Cutadapt (Version 4.4) was used to match and trim both forward and reverse primer sequences to prevent interference with subsequent analyses (Martin 2011). Quality control was performed using fastp (Version 0.23.2) (Chen et al. 2018), which removed reads shorter than 200 bp, had low quality score ≤ Q20, containing ambiguous bases, or failing to match the primer or barcode sequences. The remaining high‐quality sequences were retained as clean reads for downstream analysis.

The DADA2 plugin within QIIME2 (Version 2023.8) was used for denoising and chimera removal (Bolyen et al. 2019), generating high‐resolution amplicon sequence variants (ASVs) and a feature table. Rarefaction curves were generated using QIIME2. In order to reduce the bias caused by uneven sequencing depth across samples, the ASV feature table was rarefied to 20,000 sequences per sample. To identify the taxonomic information of plant sequences in the samples, we downloaded *rbcL* sequences from the GenBank and Barcode of Life Data System (BOLD) databases and constructed a local DNA reference database by integrating them with a local plant list. QIIME2 was used to compare the obtained ASVs with the local DNA reference database to obtain species annotations for each ASV. The analysis was conducted following the QIIME2 tutorial, along with customized program scripts (https://docs.qiime2.org/2019.1/) (Bokulich et al. 2018). The samples with the least amount of data were homogenized, and QIIME2 was used to calculate the Alpha diversity and Beta diversity, which were visualized using the ggplot2 package (Version 3.5.1) in R (Version 4.3.2). UpSet and Venn network diagrams were utilized to visualize the shared and unique ASV counts across different populations. The UpSet plot was generated using ImageGP 2.0 (https://www.bic.ac.cn/BIC/#/) (Chen et al. 2024), whereas the Venn network was visualized with EVenn (http://www.ehbio.com/test/venn/#/) (Yang, Chen, et al. 2024). Principal coordinate analysis (PCoA) and nonmetric multidimensional scaling (NMDS) on the basis of the Bray–Curtis distance matrix were computed and visualized using the ade4 (Version 1.7–22) and ggplot2 packages in R. The Levins niche width index was calculated on the basis of species richness using the SPAA (Version 0.2.2) package. Furthermore, we identified significant differences in the representative ASVs of dietary features among different populations (*p* < 0.05) using Linear Discriminant Analysis Effect Size (LEfSe) with a threshold logarithmic LDA score of 2.0 (Segata et al. 2011).

### 2.4 Statistical Analysis

The experimental data are expressed as the mean ± SD. The Kruskal–Wallis H tests were performed to compare the differences in Alpha and Beta diversity among different populations, *p < 0.05, **p < 0.01, and ***p < 0.001.

## 3 Results

### 3.1 Overall Description of *rbcL* Metabarcoding Sequencing

**FIGURE 3:**
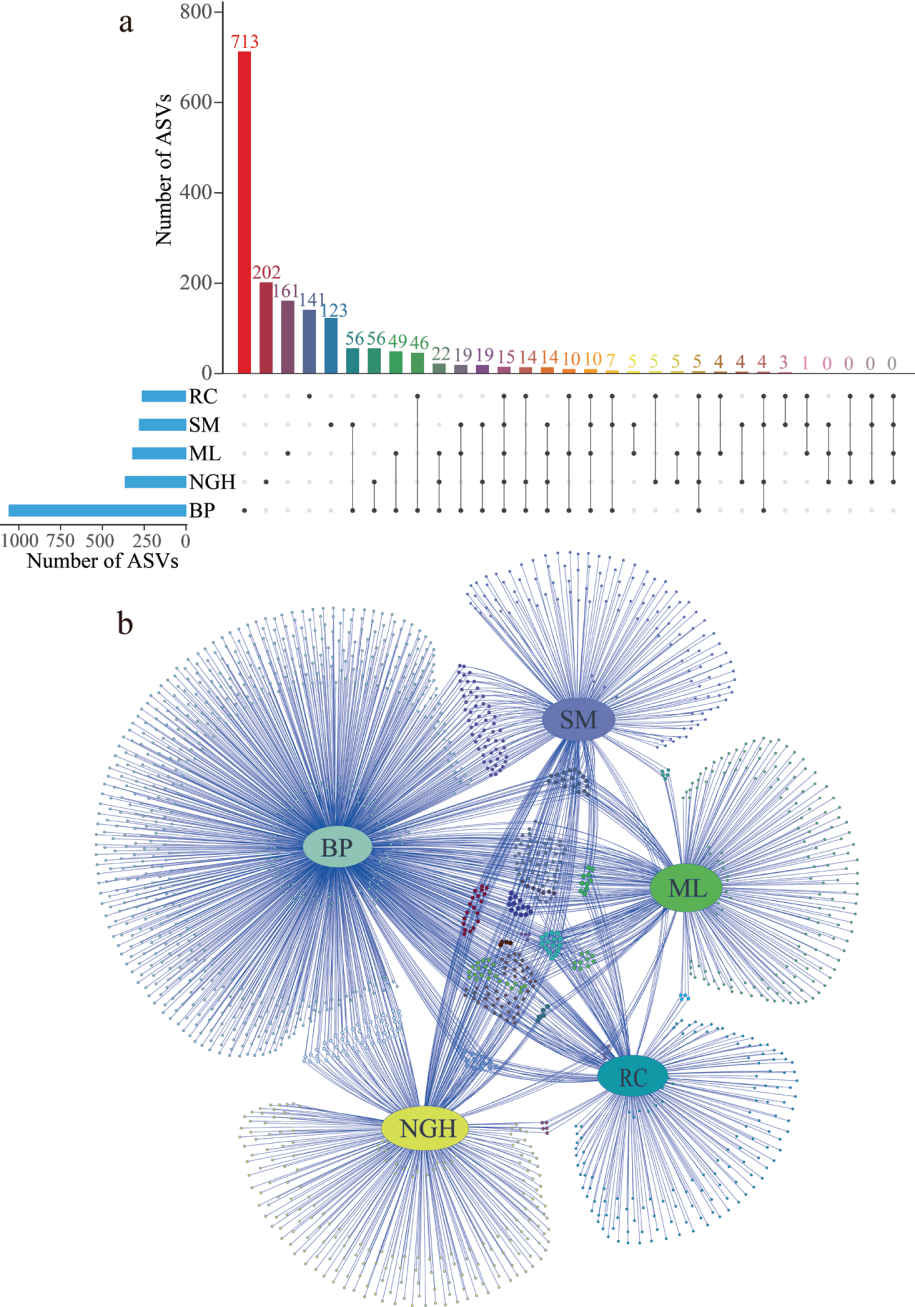
The UpSet plot and Venn network diagram of the food composition of different populations of Asian elephants. (a) The UpSet plot shows the distribution of amplicon sequence variants (ASVs) across different populations. The bar chart at the bottom left displays the number of ASVs in each population. Each point at the bottom right represents a population, with lines connecting the points indicating intersections between the populations. The bar chart and numbers at the top show the number of ASVs at the intersections of different populations. (b) The Venn network diagram shows the correspondence of ASVs between different populations.

Using high‐throughput sequencing of plant chloroplast *rbcL* metabarcoding, a total of 11,163,520 raw reads were generated from 107 fecal samples (Table S2). After quality filtering and noise reduction, 10,846,112 clean reads were obtained, with an average of 101,366 clean reads per sample and a mean read length of 218 bp (Table S2). All clean reads were clustered at 100% similarity to generate 1875 ASVs (Table S3). The numbers of ASVs in BP, SM, ML, NGH, and RC populations were 836, 120, 161, 201, and 139, respectively, with 15 ASVs shared among them (Figure 3). The alignment of the ASVs against the database resulted in the annotation of plant‐based dietary information to eight classes, 43 orders, 77 families, and 154 genera (Table S3).

### 3.2 Dietary Compositions at Different Populations

**FIGURE 4:**
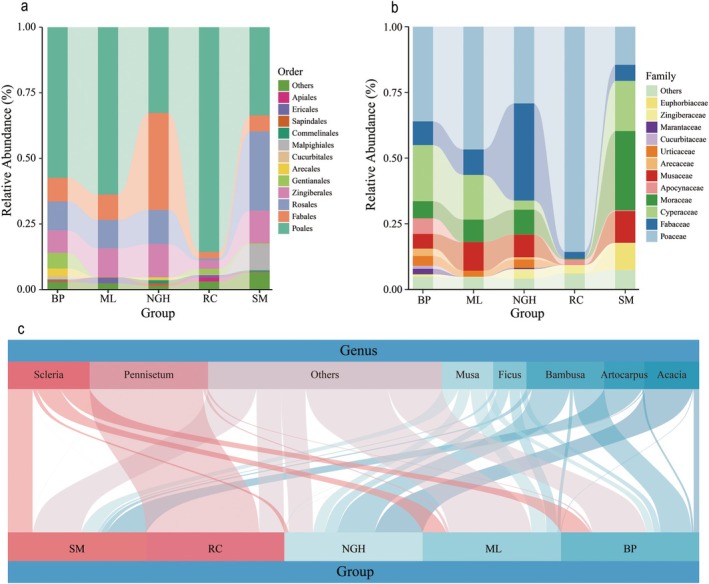
Dietary composition of Asian elephants across different populations in China. (a) The stacked bar plot shows the relative abundance of main diet types at the order level within each group. Different colors represent different orders, and lower abundance taxa are grouped together as “Others”. (b) The stacked bar plot shows the relative abundance of main diet types at the family level within each group. Different colors represent different families, and lower abundance taxa are grouped together as “Others”. (c) The Sankey plot displays the relative abundance of main diet types at the genus level within each group. The width of the connecting bars indicates the relative abundance of diet types at genus levels in different groups, and lower abundance taxa are grouped together as “Others”.

The dietary composition analysis revealed significant differences among the five populations (Figure 4 and Tables S4–S6). At the order level, the primary food sources for Asian elephants include Poales, Fabales, Rosales, Zingiberales, Gentianales, and Malpighiales, which together constitute more than 90% of their diet (Table S4). Specifically, Poales dominated the diet across all populations, accounting for 57.43% in BP, 33.63% in SM, 63.77% in ML, and 32.70% in NGH among wild populations, while reaching 85.73% in RC. Fabales was the second most abundant food source in NGH, accounting for 36.96%, but contributed only 6.10%–9.67% in other wild populations. Rosales was the second most abundant food source in SM, accounting for 30.21%, but contributed only 10.82%–12.99% in other wild populations. Zingiberales exhibited relatively consistent contributions across populations, ranging from 8.54% to 12.62%, except for a lower contribution of 3.14% in RC. Notably, Malpighiales accounted for 9.92% of the diet in SM, but less than 1% in the other populations (Figure 4a and Table S4).

At the family level, Poaceae was the dominant family in the BP, ML, and RC, with relative abundances of 36.03%, 46.74%, and 85.73%, respectively. However, Fabaceae (36.96%) and Moraceae (30.17%) were the most dominant families in the NGH and SM, respectively. Specifically, the forage composition in RC was relatively simple, with Poaceae accounting for as much as 85.73%, followed by Zingiberaceae (3.14%) and Fabaceae (2.43%). In BP and ML, Poaceae (relative abundance 36.03% in BP, 46.74% in ML), Cyperaceae (21.39%, 17.03%), Moraceae (6.50%, 8.52%), and Fabaceae (9.01%, 9.67%) dominated the diet. In contrast, the most dominant families in SM were Moraceae (30.17%), Cyperaceae (19.11%), Poaceae (14.51%), and Musaceae (12.05%). Fabaceae, Poaceae, Moraceae, and Musaceae were the dominant families in NGH, with relative abundances of 36.96%, 29.20%, 9.44%, and 8.60%, respectively (Figure 4b and Table S5).

At the genus level, the most dominant food source in RC was *Pennisetum*, contributing 81.81% of the diet. In SM, *Scleria*, *Artocarpus*, and *Musa* were the predominant genera, with relative abundances of 17.89%, 27.51%, and 12.05%, respectively. *Acacia* and *Bambusa* were the most dominant genera in NGH, contributing 34.72% and 27.08% of the diet, respectively. Similarly, the most dominant genera in BP were *Acacia* (39.90%), *Bambusa* (22.58%), and *Scleria* (21.18%). In contrast, *Scleria*, *Musa*, and *Ficus* were the most dominant genera in ML, with relative abundances of 17.01%, 10.87%, and 7.75%, respectively (Figure 4c and Table S6). Across all populations, a large number of genera with low relative abundances were detected, collectively accounting for 14.02% to 26.83% of the total diet composition (Table S6).

### 3.3 Alpha Diversity and Niche Width of Diets at Different Populations

**FIGURE 5:**
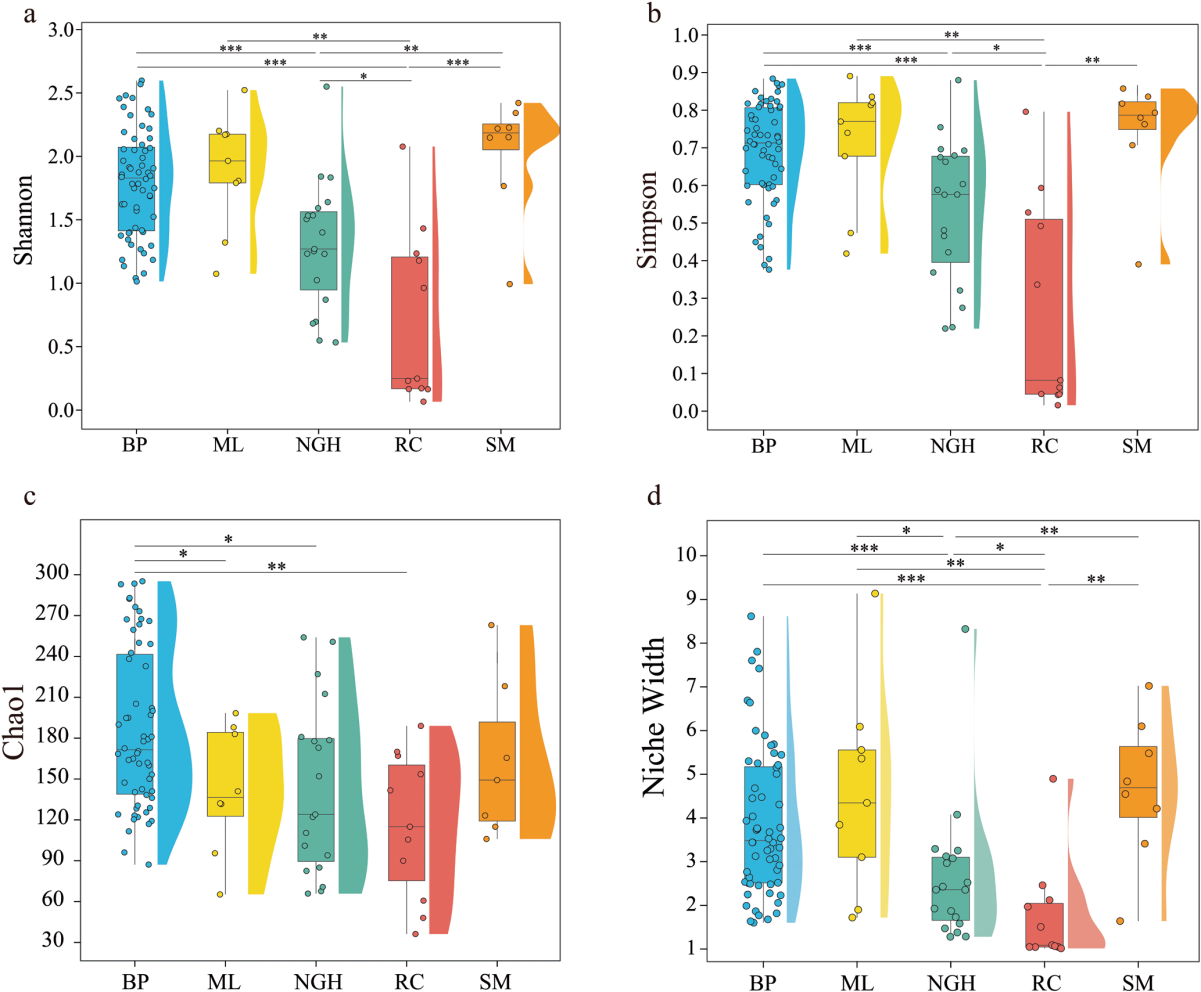
Dietary Alpha diversity and niche width of the Asian elephant across different populations in China. (a–c) Box plots respectively display the comparison of Alpha diversity differences in dietary composition across different populations, with (a) showing the Shannon index, (b) showing the Simpson index, and (c) showing the Chao1 index. (d) The Levins' niche width index, calculated on the basis of species abundance values. Significant differences detected by the Kruskal–Wallis H test, “*” indicates a significant difference between two groups, *p < 0.05, **p < 0.01, and ***p < 0.001.

Alpha diversity indicates intergroup differences in the diet diversity between areas of Asian elephants (Figure 5). The Shannon and Simpson indices show that SM has the highest diversity, whereas RC has the lowest diversity (Figure 5a,b). There are significant differences between RC and the other groups (*p* < 0.05 or *p* < 0.01), with extremely significant differences between RC and BP (*p* < 0.001), whereas no significant difference is observed between BP and ML (*p* > 0.05). Additionally, there is a significant difference in the Shannon index between NGH and SM (*p* < 0.01), but no significant difference in the Simpson index (Figure 5a,b). The Chao1 index indicates that BP has the highest dietary richness, whereas RC has the lowest. Significant differences in the Chao1 index are observed only between BP and ML (*p* < 0.05), NGH (*p* < 0.05), and RC (*p* < 0.01) (Figure 5c).

The niche width index indicates that the SM and ML have the highest niche widths, with 4.66 ± 1.66 and 4.56 ± 2.31, respectively, with no significant difference between them (*p* > 0.05). The RC group has the lowest niche width (1.75 ± 1.17), which differs significantly from all other groups (*p* < 0.05 or *p* < 0.01). The niche width of the NGH group, with 2.65 ± 1.59, is also significantly different from all other groups (*p* < 0.05 or *p* < 0.01). The niche width of the BP group, with 3.89 ± 1.71, shows no significant difference compared to the SM and ML groups (Figure 5d and Table S7).

### 3.4 Beta Diversity of Diet at Different Populations

**FIGURE 6:**
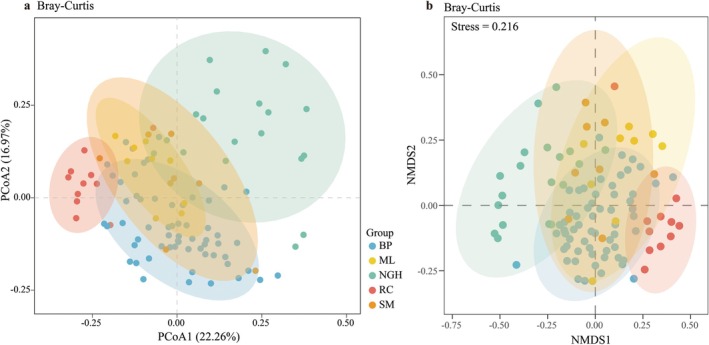
Dietary Beta diversity of Asian elephants across different populations in China. (a) Principal Coordinate Analysis (PCoA) of the Beta diversity in dietary composition across different populations on the basis of Bray–Curtis distance. Ellipses represent 95% confidence intervals for each population. (b) Nonmetric multidimensional scaling (NMDS) analysis of Beta diversity in the dietary composition across different populations on the basis of Bray–Curtis distance. Ellipses represent 95% confidence intervals for each population.

PCoA and NMDS on the basis of Bray–Curtis distance for beta diversity analysis revealed significant dietary differences among certain regions of Asian elephants (Figure 6). Notably, the captive population RC was completely separated from all wild populations, indicating that its dietary composition differed significantly from that of the wild populations. Additionally, NGH within the wild populations exhibited differences in dietary composition compared to other populations. On the basis of PCoA and NMDS analyses, the results showed a high similarity in dietary composition among the SM, ML, and BP populations (Figure 6a,b).

### 3.5 Dietary Differences at Different Populations

**FIGURE 7:**
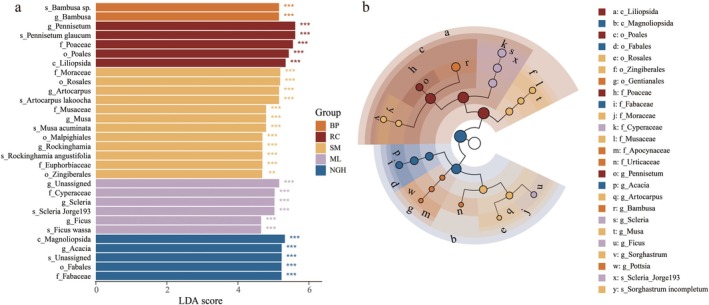
LEfSe analyses identified differentially abundant dietary taxa across five populations of Asian elephants. (a) The differential represented ASVs, and the LDA scores among diets of different populations were determined by LEfSe analysis. The letters preceding the ASVs represent the taxonomic levels (p for phylum, c for class, o for order, f for family, and g for genus). (b) The cladogram illustrates the phylogenetic relationships among significantly different dietary taxa across groups.

LEfSe analysis revealed that the SM group contained several food taxa with high abundance, including the orders Rosales, Malpighiales, and Zingiberales, the families Moraceae, Musaceae, and Euphorbiaceae, and the genera *Artocarpus*, *Musa*, and *Rockinghamia* (Figure 7). In the ML, significantly more abundant forage taxa included the family Cyperaceae and the genera *Scleria* and *Ficus*. In the NGH, the primary taxa included the class Magnoliopsida, along with the order Fabales, the family Fabaceae, and the genus *Acacia*. In the BP, the primary taxa included the genus *Bambusa* and its species *Bambusa* sp. In the captive population RC, the significantly different species was *Pennisetum glaucum* from the order Poales and family Poaceae, with a proportion as high as 81.81% (Figure 7 and Table S6).

## 4 Discussion

In this study, we analyzed chloroplast *rbcL* DNA metabarcoding data from 107 fecal samples of Asian elephants from different populations in China during the dry season, providing a detailed description of the dietary composition and diversity of Asian elephants. The results indicated significant dietary differences among the populations, which may be associated with differences in habitat plant diversity and availability.

### 4.1 Dietary Composition and Diversity of Asian Elephants in China

Asian elephants have an extensive diet, which is influenced by various factors such as the types and availability of plant species in their habitats, with their food choices ranging from several dozen to over a hundred plant species (Peng et al. 2023). This study indicates that the primary dietary sources of Asian elephants include Poaceae, Fabaceae, Cyperaceae, and Moraceae, consistent with findings from previous research (Jiang et al. 2019; Peng et al. 2023). However, variations in plant types and availability across different regions may contribute to differences in the dietary composition proportions among populations. For example, the dietary compositions of the BP and ML populations are similar, primarily consisting of Poales, Fabales, and Rosales (Figure 4). This similarity can be attributed to the comparable geographical environments and vegetation types in the two regions. In contrast, there is a significant dietary difference between the NGH and SM populations. The proportion of Fabales in the NGH population reaches 36.96%, whereas the SM population mainly consists of Rosales (30.21%) and Poales (33.62%) (Figure 4). This difference is primarily attributed to the unique geographical location of NGH, which has fostered the development of tropical and subtropical monsoon evergreen broadleaf forests (Liu et al. 2016; Sun et al. 2021), whereas SM is dominated by evergreen broadleaf forests (Chen et al. 2022).

Our study found that the NGH population consumes a large amount of Bambusa and Acacia, which differs from other populations (Figure 4). Previous research has indicated that the entire area of the Nangunhe national nature reserve is covered with bamboo forests, shrublands, and grasslands (Sun et al. 2021), which may be one of the reasons for the dietary preferences of the NGH population. Furthermore, our dietary findings for the NGH population differ from those of a previous study. In our research, the diet of the NGH population primarily consists of Fabaceae (36.96%), Poaceae (29.20%), and Moraceae (9.44%), whereas the study by Peng et al. (2023) found that the diet of the NGH population was mainly composed of Poaceae (47.69%), Moraceae (21.25%), and Musaceae (11.24%), with only 3.45% from Fabaceae. The difference may be attributed to the variation in sampling times between the two studies and changes in the availability of plant species in their habitats, and it also reflects the plasticity of the Asian elephant's diet.

The Alpha and Beta diversity of Asian elephants differs significantly across regions (Figures 5, 6), indicating that their diet is influenced by the food availability in their habitats. Food availability is one of the key factors affecting the survival of wildlife (Goldberg et al. 2020; Huang et al. 2021). In our study, the dietary diversity and richness of different populations are closely related to the availability of food in their habitats, with these differences primarily determined by the accessible food resources. For example, because of the close geographical proximity and similar vegetation types of the BP and ML populations, there is no difference in their dietary diversity and niche widths, with only differences in richness (Figure 5). This is similar to the findings in other species, such as the significant dietary composition differences observed in Eurasian otters ( *Lutra lutra* ) across different regions (Fu et al. 2024). In addition, we found that the dietary niche widths of BP, ML, and SM are significantly wider than that of NGH (Figure 5d), as these three regions have higher elephant population densities, and the expansion of forest canopy coverage has reduced the availability of understory food resources (Yu et al. 2023). Particularly in Xishuangbanna, the Asian elephant population accounts for 95% of the total in China (Cao et al. 2023; Wang et al. 2021), whereas there are only about 20 elephants in the NGH region (Sun et al. 2021). The higher population density and scarcity of food resources in these areas have led the Asian elephants to require a wider ecological niche to obtain food.

### 4.2 Implications for Conservation Management

We found that the diet of the captive RC population is dominated by Pennisetum, accounting for 81.81% (Figure 4c), which is significantly higher than that of the wild populations, and its diet has the lowest diversity and niche widths (1.75 ± 1.17) (Figure 5). This inappropriate food supplementation affects digestion rates and increases the risk of disease transmission (Couch, Wise, et al. 2021; Putman and Staines 2004; Zheng et al. 2024). To address these issues, it is essential to optimize the food structure in captive management by diversifying the diet to better mimic natural feeding patterns, thereby reducing disease risks and improving overall health.

Currently, the establishment of ecological corridors and food source bases for Asian elephants has become a crucial measure to mitigate human–elephant conflicts and protect Asian elephants and their habitats (Li et al. 2023; Liu et al. 2017; Zhang et al. 2024). This study provides insights into variations in dietary diversity and geographical distribution among different Asian elephant populations in China. These findings provide essential scientific evidence and practical guidance for optimizing vegetation in ecological corridors and constructing food source bases for Asian elephants in China. In the management of wild populations, the construction of ecological corridors and food source bases should be scientifically planned on the basis of regional dietary preferences and ecological needs. Priority should be given to restoring key plant groups such as Poaceae, Fabaceae, Cyperaceae, Musaceae, and Moraceae to ensure the primary food sources for Asian elephants. At the same time, the construction of food source bases in different regions should be tailored to local elephant herd dietary characteristics. For example, in the NGH region, the planting of Fabaceae plants can be increased, whereas in the SM region, more Moraceae and Musaceae plants should be introduced.

Diet is one of the key factors influencing the structure of an animal's gut microbiota (Couch, Stagaman, et al. 2021; Yang, Deng, et al. 2024). As typical hindgut fermenters, Asian elephants rely on complex gut microbial communities to ferment cellulose, thereby obtaining the nutrients necessary for their vital activities (Bereswill et al. 2014; Bo et al. 2023; Zhang et al. 2023). Our previous study demonstrated a significant correlation between gut microbial composition and diet in Asian elephants (Guo et al. 2025). Notably, the RC population, with a highly monotonous diet, exhibited reduced microbial diversity and associated negative impacts on gut health. Therefore, in future conservation efforts, long‐term monitoring of the diet and dynamic gut microbiota of Asian elephants is essential. This will help assess the elephant herd's adaptation to habitat restoration and provide scientific evidence for optimizing conservation strategies.

## 5 Conclusions

This study utilized DNA metabarcoding technology to analyze the dietary information of different Asian elephant populations in China, revealing differences in dietary composition and diversity. The results showed that the diet of Asian elephants primarily consists of Poaceae, Fabaceae, Cyperaceae, Musaceae, and Moraceae plants, with significant geographical differences. This indicates that Asian elephants exhibit flexible feeding strategies, and their dietary structure is influenced by habitat plant resources and environmental conditions. Additionally, the diet of captive populations, which is dominated by a single plant species, may have adverse effects on their health. Therefore, it is urgent to explore captive care models that better mimic natural environments to improve health conditions and enhance management effectiveness. This study not only deepens our understanding of the foraging ecology of Asian elephants but also provides new perspectives and scientific evidence for optimizing the food structure of captive populations and the development of food resource bases.

However, the samples in this study were predominantly obtained during the dry season, as sample collection during the rainy season is particularly challenging. This limited sampling may restrict the ability to fully capture seasonal variations in dietary composition throughout the year. Moreover, this study focused exclusively on plant composition and diversity, without analyzing the nutritional content of different food items, thereby limiting insights into the potential impacts of diet on population health. Additionally, although fecal DNA metabarcoding is a powerful non‐invasive tool for studying animal diets, it has certain limitations that should be carefully considered. Primer specificity can cause amplification bias, resulting in the under‐ or overrepresentation of certain taxa (Alberdi et al. 2019; Ando et al. 2020; Sato 2025). Incomplete reference databases may further limit taxonomic resolution, particularly for poorly characterized plant groups (Sato 2025). Although rbcL‐based DNA metabarcoding provides valuable information on plant taxa, it has limitations in distinguishing closely related species and cannot achieve precise species‐level identification.

## Author Contributions

**Qiang Guo:** conceptualization (equal), data curation (equal), investigation (equal), resources (equal), writing – original draft (equal). **Wenping Zhang:** data curation (equal), supervision (equal), writing – original draft (equal), writing – review and editing (equal). **Xu Li:** data curation (equal), funding acquisition (equal), investigation (equal), writing – original draft (equal). **Bin Wang:** resources (equal), software (equal). **Chaoyong Xiong:** resources (equal), software (equal). **Yuan Tian:** resources (equal), software (equal). **Tingting Luo:** resources (equal), software (equal). **Weibin Wang:** investigation (equal), supervision (equal), writing – original draft (equal), writing – review and editing (equal). **Jielong Zhou:** data curation (equal), formal analysis (equal), writing – original draft (equal), writing – review and editing (equal).

## Conflicts of Interest

The authors declare no conflicts of interest.

## Supporting information


**Data S1:** ece372398‐sup‐0001‐DataS1.xlsx.

## Data Availability

The DNA metabarcoding data generated in this study have been deposited in the NCBI Sequence Read Archive (SRA) under the accession number PRJNA1245591.
